# The Biological and Biophysical Properties of the Spider Peptide Gomesin

**DOI:** 10.3390/molecules23071733

**Published:** 2018-07-16

**Authors:** John D. Tanner, Evelyne Deplazes, Ricardo L. Mancera

**Affiliations:** School of Pharmacy and Biomedical Sciences, Curtin Health Innovation Research Institute and Curtin Institute for Computation, Curtin University, GPO Box U1987, Perth WA 6845, Australia; john.d.tanner@student.curtin.edu.au (J.D.T.); r.mancera@curtin.edu.au (R.L.M.)

**Keywords:** toxins, peptides, spider peptides, peptide-membrane interactions, anti-microbial peptides, anti-cancer peptides, structure-activity relationship, rational drug design

## Abstract

This review summarises the current knowledge of Gomesin (Gm), an 18-residue long, cationic anti-microbial peptide originally isolated from the haemocytes of the Brazilian tarantula *Acanthoscurria gomesiana.* The peptide shows potent cytotoxic activity against clinically relevant microbes including Gram-positive and Gram-negative bacteria, fungi, and parasites. In addition, Gm shows in-vitro and in-vivo anti-cancer activities against several human and murine cancers. The peptide exerts its cytotoxic activity by permeabilising cell membranes, but the underlying molecular mechanism of action is still unclear. Due to its potential as a therapeutic agent, the structure and membrane-binding properties, as well as the leakage and cytotoxic activities of Gm have been studied using a range of techniques. This review provides a summary of these studies, with a particular focus on biophysical characterisation studies of peptide variants that have attempted to establish a structure-activity relationship. Future studies are still needed to rationalise the binding affinity and cell-type-specific selectivity of Gm and its variants, while more pre-clinical studies are required to develop Gm into a therapeutically useful peptide.

## 1. Introduction

Gomesin (Gm) is a cationic anti-microbial peptide (AMP) that was originally isolated from the haemocytes of the unchallenged Brazilian tarantula *Acanthoscurria gomesiana* [1]. The peptide is part of the innate immune system of the spider and is released during microbial infection [2]. Like many other AMPs, Gm shows cytotoxic activity against a wide range of pathogens, including clinically-relevant Gram-positive and Gram-negative bacteria, fungi and yeast, as well as demonstrating anti-malarial [3,4], anti-cryptococcal [5], and anti-*Leishmania* activity [1,3,6,7,8,9,10,11]. Gm also has in-vivo anti-cancer activity in a mouse model of melanoma [12], and in-vitro activity against a number of other human cancers [3,9,12,13,14,15,16,17]. In addition, Gm shows cytotoxic and anti-proliferative activity against devil facial tumour disease [18], a “parasitic” form of cancer that threatens the extinction of the Tasmanian devil (*Sarcophilus harrisii*), a unique Australian animal. A number of studies have successfully used different types of chemical modifications, such as cyclisation and/or amino acid substitution, to increase the anti-microbial or anti-cancer properties of Gm [3,8,9,18]. This wide range of cytotoxic activity, combined with its high serum stability [3,6,8,9] and moderate levels of haemolysis [1,6,7,8,9], make Gm of interest for the development of therapeutics to treat microbial infections and cancer. 

Most AMPs and anti-cancer peptides (ACPs), including Gm, exert their cytotoxic activity via membrane permeabilisation. A number of mechanisms have been proposed to explain the activity of AMPs/ACPs, which include, but are not limited to, the formation of pores, a ‘carpet’ (detergent-like) mechanism, and a ‘sinking raft’ model. Despite extensive research on a large number of AMPs/ACPs, the molecular details of their mechanism of action remain poorly understood [19,20,21,22,23,24]. Some modifications that alter the physico-chemical properties of Gm result in changes to its activity yet, other, similar modifications, have little to no effect. This clearly highlights our limited understanding of the molecular mechanism of action of Gm (and of AMPs/ACPs in general).

The ability of Gm to permeabilise cell membranes has been established both in leakage experiments using lipid vesicles [9,25,26,27,28] and in whole-cell experiments with bacterial [9] and cancer cells [9,12]. Furthermore, it has been demonstrated that Gm shows a preference for membranes that contain negatively charged lipids [3,9,26,27]. This is consistent with the view that the selectivity of AMPs/ACPs for bacterial and cancer cells is mainly governed by electrostatic interactions between the cationic peptides at the negatively charged lipids that are found at increased concentrations in bacterial and cancer cells compared to healthy human cells [19,21,29,30,31].

A range of techniques has been used to elucidate the structure and molecular mechanism of action of Gm. The structure of Gm in aqueous and cell membrane (or membrane-mimicking) environments has been studied using nuclear magnetic resonance (NMR) and circular dichroism (CD) spectroscopy [7,32]. The membrane-binding properties and peptide-lipid interactions have been investigated using surface plasmon resonance (SPR) [3,9], electron paramagnetic (EPR) spectroscopy, and isothermal calorimetry (ITC) [25,26,28]. Other studies have focused on the intra-cellular and/or ‘downstream’ effects of Gm [12,13,16,17,18,33]. In an effort to establish a structure-activity relationship, many of these studies have not only used native (wild-type) Gm, but also a large number of Gm variants with altered structural and/or physico-chemical properties. In fact, since the discovery of Gm, more than 40 variants have been designed and tested. This review aims to summarise the current knowledge of Gm, with a particular focus on its biophysical characterisation and that of its variants. The remainder of this review is organised as follows: Section 2 summarises the in-vitro activity of Gm against the most commonly tested microbes and cancer cell lines, the few reported in-vivo activity studies, as well as the haemolytic activity of the peptide. Section 3 and Section 4 review the studies primarily focused on the characterisation of the structure of Gm and the relationship to its serum stability. This is followed by an overview of the membrane-binding properties (Section 5) and the leakage activity and permeabilisation properties (Section 6) of Gm. Section 7 is focused on the insights gained from experiments with Gm variants to understand the role of peptide hydrophobicity and charge in cytotoxicity. Finally, Section 8 describes the intra-cellular actions of Gm. The review concludes with a summary of our current knowledge of Gm.

## 2. Cytotoxic and Haemolytic Activity of Gomesin

### 2.1. Anti-Microbial Activity

The original study reporting the isolation, synthesis, and biochemical characterisation of Gm included testing the cell viability on more than 40 microorganisms, including Gram-positive and Gram-negative bacteria, filamentous fungi and yeasts, and the eukaryotic parasite *Leishmania amazonensis* [1]. Since then, the anti-microbial activity of Gm has been confirmed by a number of independent studies [3,7,8,9,11,34]. Table 1 summarises the minimum inhibitory concentrations (MIC) for the most commonly-tested microorganisms reported in these studies. The most sensitive bacterial strains are from *Escherichia coli, Pseudomonas aeruginosa*, and *Staphylococcus aureus*, followed *by Klebsiella pneumoniae.* Gm is also very effective against clinically-relevant yeast such as *Candida albicans* and *Cryptococcus neoformans.*

To the best of our knowledge, only two in-vivo studies of the anti-microbial activity of Gm have been published. Rossi et al. investigated the efficacy of Gm against disseminated and vaginal candidiasis in mice infected with the clinical strain of *C. albicans* (isolate 78) [34]. In both types of infections, Gm effectively reduced fungal burden compared to the control group, and did so at lower concentrations than fluoconazole (a commonly used anti-fungal agent to treat yeast infections in humans and animals). Gm also increased the concentration of three cytokines (IL-6, TNFα and IFNγ), indicating that it also possesses immunomodulatory activity. A synergistic effect of Gm and fluoconazole was observed in-vitro for two isolates of *C. albicans* (isolate 78 and ATCC90028), as assessed by reduced MIC values for the two compounds compared to single compound MIC. However, in-vivo, this synergistic effect was only observed in immunocompromised mice. Moreira et al. investigated the use of Gm as an anti-plasmodium agent against the asexual, sexual, and pre-sporogonic forms of *Plasmodium falciparum* and *Plasmodium berghei* [4]. Gm impaired the development of both species without any adverse effect on the survival, fecundity, and fertility of the infected mosquitoes.

### 2.2. Anti-Cancer Activity

The in-vitro anti-cancer activity of Gm has been demonstrated in a number of studies using a range of murine and human cancer cell lines [3,9,12,13,14,15,16], as well as cells from devil facial tumour disease (DFTD) [18]. Table 2 summarises the anti-cancer activity of Gm on the most commonly tested cell lines. For most tested cell lines, the values from the different studies show acceptable agreement, with the exception of HeLa cells, that show IC_50_/CC_50_ values ranging from 8.0 μM to 72.7 μM. A comparison of the experimental protocols did not show any apparent differences that would explain the variations in IC_50_/CC_50_ values, and no explanation for the discrepancies was provided by the authors. From the data in Table 2, it is evident that Gm is most effective against several forms of melanoma and leukaemia, but less so against cervical cancer cells. While it has been shown that the increased levels of phosphatidylserine (PS) in cancer cells are an important contribution to the selective cytotoxic activity of AMPs/ACPs towards cancer cells [19,30,35,36,37,38], this can only partially explain why many AMPs/ACPs, including Gm, show marked differences in activity between different types of cancer cells [19]. The cell-type specific activity of Gm is likely dependent on additional factors such as differences in membrane composition other than PS (e.g., gangliosides, heparan sulfate, levels of cholesterol) [19,38,39], and/or intra-cellular effects (see also Section 8).

To the best of our knowledge, there has only been one in-vivo study on the anti-cancer activity of Gm, reported by Rodrigues et al. [12]. This study investigated the efficacy of Gm in a topical treatment of subcutaneous murine melanoma. A Gm-containing cream was applied to animals with established tumours. After 4 weeks, the treated animals showed a significant delay in tumour growth and increased survival times compared to control animals. Using a series of in-vitro experiments with the same B16 F10 melanoma cells, the authors further demonstrated that the anti-tumour activity was due to a direct effect on cancer cells.

### 2.3. Haemolytic Activity

Since the main mechanism of cytotoxic activity of Gm is based on the permeabilisation of cell membranes, it is not surprising that Gm shows haemolytic activity (it causes lysis of human erythrocytes). For any AMP/ACP to be used as a therapeutic agent, its haemolytic activity needs to be significantly lower than its cytotoxic activity on the target cell. This means that the concentration required to cause a specific level of haemolysis (e.g., HD10 to obtain 10% of haemolysis) should be higher than the MIC or IC_50_/EC_50_ for the bacterial or cancer cell of interest. Several studies have assessed the haemolytic activity of Gm by determining the percentage of haemolysis as a function of peptide concentration [1,6,7,8,9,10]. Machado et al. [10] reported a value of 0.39 μM for the HD10 of Gm. Although none of the other studies determined the value of HD10, in most cases it can be estimated from the data reported: the HD10 of Gm ranges from 0.39 μM to approximately 1.0 μM [1,6,7,8]. Furthermore, even concentrations as high as 100 μM did not induce haemolysis to an extent higher than approximately 40–45%. Modifications of the peptide that significantly reduce haemolysis also reduce its antimicrobial and anti-cancer activity (and most of the time, these modifications also reduce serum stability, see Section 4). A more successful approach is to find modifications that increase the activity against target cells (i.e., reduced MIC or IC_50_/E_50_), but that do not alter the haemolytic activity [9] (see Section 7). Due to our limited understanding of the molecular mechanism of Gm and the role of specific lipids in its selectivity towards different cell types, finding such mutations is, however, not straightforward.

## 3. Structure

**Figure 1:**
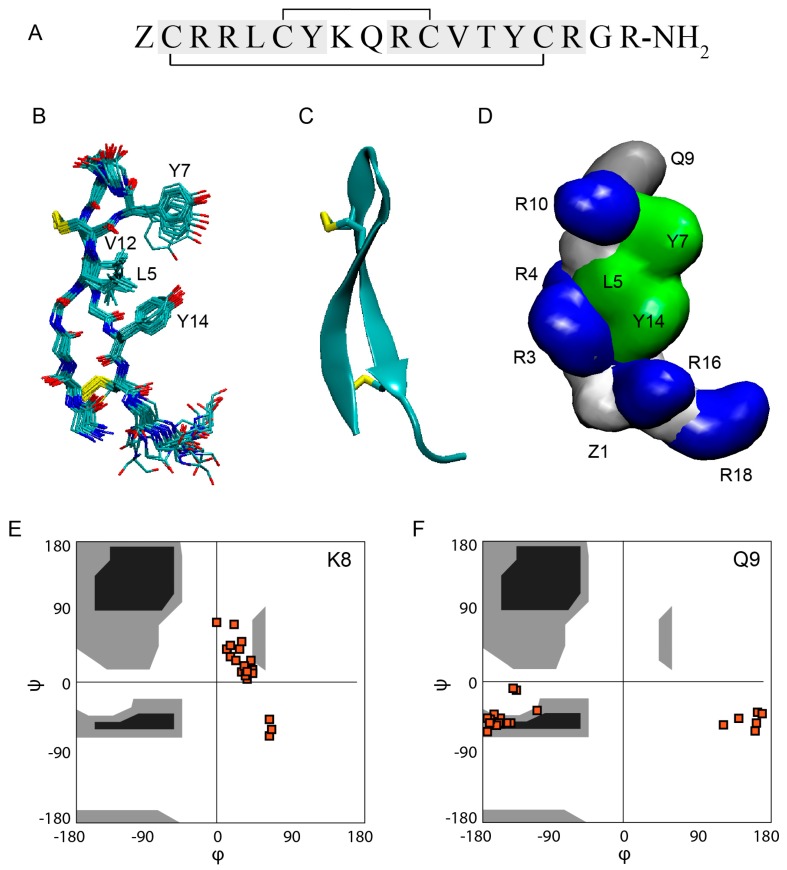
Structure of Gomesin. (**A**) Sequence of Gm with disulfide bond connectivity and the residues that form the β-strands highlighted in grey. Z = pyroglutamic acid; (**B**) Backbone superposition of the 20 final structures in the NMR ensemble collected at 5 °C by Mandard et al. [32] (PDB code 1KFP) and the sidechains of the residues that form the hydrophobic face (L5, Y7, V12, Y14). The disulfide bonds are shown in yellow; (**C**) Ribbon representation of the β-hairpin-like structure of Gm; (**D**) Surface representation of Gm coloured by residue type, with non-polar (hydrophobic) residues shown in green, polar residues in grey and basic residues in blue; (**E**,**F**) Ramachandran plots of the backbone dihedral angles in K8 and Q9 from the 20 structures of the NMR ensemble collected at 5 °C by Mandard et al. [32].

Gm is an 18-residue peptide with the sequence ZCRRLCYKQRCVTYCRGR-NH_2_, where Z is a pyroglutamic acid (pE) and R-NH_2_ is an amidated R [1] (Figure 1A). The solution structure of Gm (PDB code 1KFP) was solved at 5 °C in 90% H_2_O/10% D_2_O at pH 3 using proton 2D NMR by Mandard et al. [32]. The structure showed that the global fold of Gm consists of two-stranded anti-parallel β-strands that are connected by a 4-residue non-canonical β-turn (Y7-K8-Q9-R10), referred to as a β-hairpin-like structure. The overlay of the 20 conformations shows the presence of rigid and well-defined β-strands and a flexible C-terminus (Figure 1B). The β-strands are connected by two inter-strand disulfide bridges (Figure 1B,C), and are further stabilised by six inter-strand backbone-backbone hydrogen bonds. Both disulfide bridges adopt right-handed conformations with torsion angles close to the low energy conformations found in disulfide bonds that bridge anti-parallel beta-strands [40,41]. The Cα-Cα distances for the two disulfide bridges are 0.38 ± 0.10 nm (C6-C11) and 0.37 ± 0.10 nm (C2-C15). As in the case of the torsion angles, these are typical distances for disulfide bridges found in anti-parallel β-strands. These distances are, however, considerably shorter than the Cα-Cα distances for disulfide bridges that connect other secondary structure motifs or β-sheets found in larger, more globular peptides/proteins [40]. Gm also exhibits an unusual β-turn. The (i,i+3) hydrogen bond that is found in classical β-turns [42] is only present in 10 of the 20 structures. Furthermore, K8 and Q9 show unusual backbone dihedral angles. As seen in the Ramachandran plots (Figure 1E,F), both residues exhibit ϕ/φ angles that are outside of the favourable region (black), or even the allowed regions (grey). The combination of ϕ/φ angles found in K8 and Q9 are not found in any other type of β-turns [42]. Whether these unusual structural features are functionally relevant is not yet known.

Gm is cationic, with an overall charge of +6 at physiological pH, resulting from the presence of five R and one K residue (Figure 1A). The C- and N-termini are neutral due to amidation and the pyroglutamic acid, respectively. Apart from positive charge, the amphiphilic nature of Gm is another feature shared with most other AMPs/ACPs. As seen in Figure 1D, residues L5, Y7, V12, and Y14 form a ‘hydrophobic face’ on one side of the peptide, while charged and polar residues in the extremities of the peptide form opposing hydrophilic regions. As discussed in more detail in Section 7, the hydrophobic face is essential for the membrane-binding and leakage activity of Gm. Comparison to other peptides revealed that Gm shows structural and sequence similarities to a number of AMPs/ACPs that adopt a β-hairpin-like fold, including protegrins from porcine leukocytes [43], adroctonin from the scorpion *Androctonus australis*, and tachylepsin and polyphemusin from horseshoe crabs [44,45]. In all of these peptides, the β-strands are connected by a 4-residue turn, and the β-sheet is stabilised by two disulfide bridges. The peptides also share a relatively large percentage of basic residues. In addition, these peptides show a similar distribution of their hydrophobic and charged/polar residues. While all of these peptides were isolated from haemocytes, a Gm analogue has recently been found in the venom of the Australian funnel-web spider *Hadronyche infensa* [18].

The first structure-activity relationship studies of Gm focused on investigating the role of the disulfide bridges and the β-sheet fold in the anti-microbial and haemolytic activity of the peptide. Fazio et al. prepared a linear variant of Gomesin (GmL) in which all four C residues were replaced by S, thus removing the disulfide bridges [7,8]. In addition, a number of monocyclic and bicyclic peptides using lactam bridges instead of disulfide bridges were tested. The authors referred to Gm with two disulfide or lactam bridges as *bicyclic*, but this should not to be confused with the later described cyclised version of Gm. The effect of these modifications on the structural fold of Gm was studied using CD spectroscopy. GmL showed a spectrum typical for unordered (random coil) structures. The bicyclic compounds were determined to adopt a β-hairpin-like fold, while monocyclic compounds tend to adopt a combination of coils and helical structures. This suggests that both bridges are required for Gm to fold into its stable secondary structure. This was further confirmed by NMR α-proton chemical shifts [7]. Comparison of the anti-microbial activity of GmL, mono- and bicyclic Gm variants to native Gm showed that removing one bridge reduces activity, while removing both bridges resulted in complete loss of activity. This indicates that the β-hairpin-like fold adopted in the presence of both bridges is required for full anti-microbial activity [8]. It is, however, not the presence of the disulfide bridges (i.e., the C residues) *per se* but rather, the stability of the fold that results from inter-residue bridges that is required for activity, since bicyclic compounds with two lactam bridges show anti-microbial activity comparable to native Gm [8]. Interestingly, replacing the C residues with T residues to increase β-strand propensity, and stabilising the β-turn with a P residue at position 9, rescued to some extent the loss of anti-microbial activity seen in the linear variants [7]. As the structure of Gm is linked to its cytotoxic activity, it is not surprising that all GmL and monocyclic variants show significantly reduced haemolysis [7,8].

**Figure 2:**
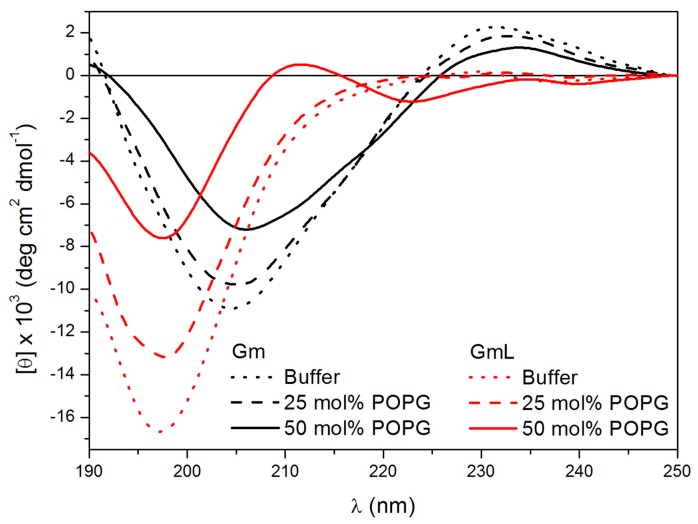
Circular dichroism spectra of Gm and GmL in an aqueous solution and in the presence of POPC with 25 mol % or 50 mol % POPG. Images reprinted from Domingues et al. [26]. Copyright (2015), with permission from Elsevier.

Since Gm works by membrane permeabilisation, the effect of lipids or membrane-mimicking environments on the conformation of Gm and its variants have been investigated. Domingues et al. used CD spectroscopy to study the conformations of native Gm and a series of W-containing Gm variants (Gm-W1, Gm-W7, Gm-W9) in the presence of anionic SDS micelles [6]. Tryptophan residues were introduced to facilitate fluorescence studies to confirm that the peptide was bound to the micelle surface. All three W-containing variants showed the same anti-microbial activity as native Gm. Furthermore, the CD spectra showed that the variants exhibit the same spectral profiles as Gm in aqueous solution, suggesting that the W-mutation did not alter the structure of the peptide. The CD spectra collected in the presence of low SDS concentration (1 mM, below the critical micelle concentration) indicate that Gm, as well as all W-containing variants, adopt the same β-hairpin-like structure as in aqueous solution. At higher SDS concentrations (15 mM, above the critical micelle concentration), the peptides undergo further structuring of the β-sheet motif. In fact, experiments from a study by Fazio et al. [8] showed that in the presence of very high SDS concentration (50 mM), even GmL, which is unstructured in aqueous solution, folds into a β-sheet similar to that of native Gm. In a subsequent study, Domingues et al. confirmed these findings by comparing the CD spectra of Gm and GmL collected in an aqueous solution and in the presence of large unilamellar vesicles (LUVs) composed of the neutral lipid POPC (1-palmitoyl-2-oleoyl-sn-glycero-3-phosphocholine) mixed with either 25 or 50 mol % of the negatively charged lipid POPG (1-palmitoyl-2-oleoyl-sn-glycero-3-phospho-(1′-*rac*-glycerol)) [26], as illustrated in Fig 2. As in the case of SDS micelles, native Gm showed a slightly more pronounced β-sheet structure in the presence of 50% POPG. The structure of GmL was determined to be very similar in an aqueous solution and in the presence of POPC LUVs containing 25% POPG, with peaks characteristic of a β-sheet structure only appearing when the amount of POPG was increased to 50% (Figure 2). These studies indicate that interactions with negatively charged lipids or detergent molecules stabilise the β-sheet fold of Gm to such an extent that the peptide can fold into a stable β-sheet structure, even in the absence of disulfide bridges.

A number of studies have investigated a cyclised version of Gm (cyclic Gm, cGm), in which the N-terminal Z (pE) residue is replaced with G and amidation at the C-terminus is removed. The resulting G and R termini are chemically ligated (‘fused’) [3,10]. The NMR structure of cGm shows the same overall fold observed in Gm, with the only difference being the removal of the disordered termini, resulting in a more structured peptide [3]. In general, the stable β-sheet fold of Gm means that, apart from alteration of the disulfide bridges, chemical modifications such as point mutations of one or more residues do not significantly alter its structure [9].

## 4. Serum Stability

Serum stability reflects the ability of a peptide to resist degradation by enzymes found in human blood serum, and is critical for its successful use as a therapeutic agent [46]. Serum stability is usually assessed by incubating a concentrated solution of a peptide with diluted human blood serum at 37 °C, and then determining the percentage of peptide remaining after one or multiple fixed-time intervals. Using this approach, a number of studies have demonstrated the high serum stability of Gm. After 6 h, at least 85% of the peptide remained detectable [7,8,10]. Even after 24 h, levels as high as 75% of the intact peptide were detectable [9], while other studies reported approximately 35% [3]. As expected [47], the serum stability of Gm is directly related to the presence of disulfide bridges [7,8,10]. Gm variants with only one disulfide bridge ([S^2-15^]-Gm, [S^6-11^]-Gm) show reduced serum stability: only 40–50% of intact peptide was detectable after 1 h of incubation [8]. When both disulfide bridges are removed, the peptide is almost completely degraded after 1 h [7,8]. Similar to anti-microbial activity, serum stability is retained upon replacement of the disulfide bridges with lactam bridges [7]. It has also been reported that cyclisation improves serum stability [48]. While cGm shows the same levels of degradation after 6 h of incubation [3], it shows higher stability after 24 h [3,9]. Cyclic variants without the two disulfide bridges were slightly more stable than GmL, but were still significantly more susceptible to degradation than native Gm or cGm [10]. Finally, introducing residues that increase β-sheet propensity and/or D-amino acids increases resistance to proteolysis [8,13], but still does not provide the same high serum stability observed with disulfide bonds. It can be concluded that for optimal serum stability, both disulfide bridges need to be present, while cyclisation can provide additional protection against degradation.

## 5. Permeabilisation of Cell Membranes and Leakage Activity

**Figure 3:**
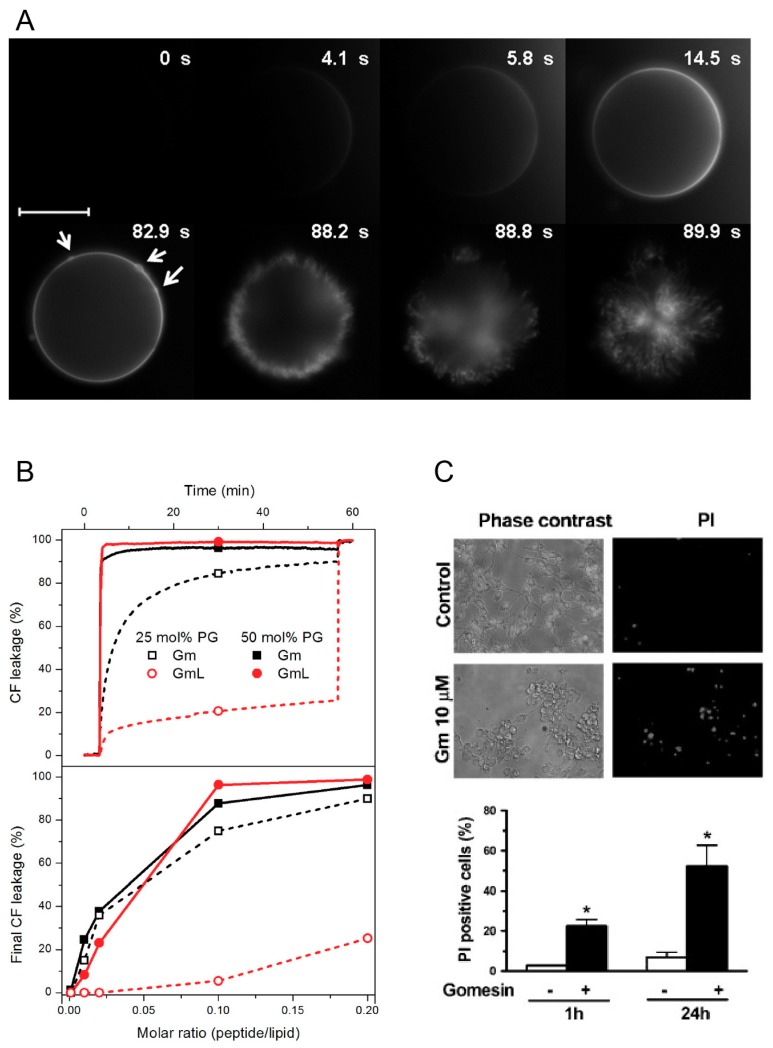
Leakage and permeabilisation activity of Gomesin (Gm). (**A**) Sequence of microscopy images for the injection of a fluorescently labelled Gomesin solution to a GUV composed of POPC/POPG (1:1). Arrows indicate the aggregation of peptide on the membrane surface. The scale bar is 20 μm. Adapted with permission from Domingues et al. [27]. Copyright 2010 American Chemical Society; (**B**) Top panel: CF (5(6)-carboxyfluorescein) leakage from LUVs composed of POPC and 25 mol % or 50 mol % POPG. Bottom panel: Percentage of CF leakage as a function of peptide-lipid ratio. Images reprinted from Domingues et al. [26]. Copyright (2015), with permission from Elsevier; (**C**) Top panel: Effect of Gm on cell viability and permeabilisation of human SH-SY5Y cells. Photomicrographs taken with phase contrast (left) and fluorescence filter (right) to visualise cells stained with prodidium iodide (PI). Bottom panel: Histograms showing a quantitative analysis of PI-positive cells with (+) and without (−) treatment of Gm. Images adapted from Soletti et al. [17]. Copyright (2010), with permission from Elsevier.

The ability of Gm to permeabilise cell membranes has been established, both in leakage experiments using lipid vesicles [9,25,26,27,28], and in whole-cell experiments with bacterial [9], yeast [5] and cancer cells [9,12]. In addition, experiments with melanoma cells have demonstrated internalisation and cellular distributions of Gm [13]. Domingues et al. were among the first to study the lytic mechanism of Gm [27]. They investigated the lytic activity of Gm and GmL using optical microscopy and giant unilameller vesicles (GUVs) made of different molar fractions of neutral POPC and negatively charged POPG (mimicking bacterial cell membranes), as well as GUVs composed of POPC with 40% cholesterol (as a model of mammalian cell membranes). To qualitatively study the lytic mechanism of Gm, the authors used two complementary approaches. First, they used microinjection of unlabelled Gm into POPC GUVs containing 1 mol % DiIC18, a lipophilic fluorescent dye that accumulates in the membrane, and thus allows visualisation of the vesicle. Second, they used microinjection of fluorescently labelled Gm (Gm-Rh) into GUVs composed of unlabelled POPC or POPC/POPG (1:1 mol/mol). In both cases, the sudden bursting of the vesicles was preceded by the local accumulation of peptides on the membrane surface (Figure 3A). The effects observed were independent of the lipid composition and peptide, and the authors concluded that this accumulation of peptides is part of the membrane-disrupting mechanism of Gm. An additional set of experiments were aimed at quantifying lytic activity by using increasing concentrations of Gm and GmL against GUVs with different membrane compositions. The ‘minimum bursting concentration’ (MBC, the lowest concentration of peptide required to burst 90% of GUVs) was determined. As expected from the low anti-microbial activity of GmL, its MBC is significantly higher compared to Gm (5.0 ± 0.5 μM for GmL, 1.5 ± 0.5 μM for Gm). The reduced lytic activity of GmL was confirmed in a subsequent study by the same authors by monitoring the peptide-induced leakage of the fluorescence probe CF (5(6)-carboxyfluorescein) entrapped in LUVs composed of POPC and 25 or 50 mol % POPG [26]. Gm induced rapid leakage of CF (within a few tens of seconds) in LUVs with 25 or 50 mol % POPG. In contrast, GmL caused slower leakage in LUVs with 50% POPG, and only mild leakage in LUVs with 25% POPG (Figure 3B). These experiments also indicated that Gm has a preference for negatively charge membranes. For both Gm and GmL, leakage increased with increasing proportion of POPG. In contrast, the MBC was determined to be about 3-fold higher for a LUV containing 40% cholesterol compared to POPC only. This preference for LUVs containing negatively charged lipids was also observed for cGm. Using CF leakage experiments, Henriques et al. reported a more than 40-fold increase of leakage for LUVs composed of POPC/POPG (80:20 mol % i.e. 4:1) compared to POPC LUVs [9]. In addition to lipid preferences, these leakage experiments demonstrated that Gm can induce membrane permeabilisation through a lipid-dependent mechanism without the involvement of cell surface receptors or other membrane proteins found in plasma membranes.

While leakage experiments with vesicles are useful to establish the concentration-dependence of the mechanism of lysis, and can provide information on lipid selectivity, it is still important to demonstrate membrane permeabilisation in actual cells. This is particularly important to characterise the selectivity of Gm for specific cancer and/or bacterial cells, as their complex membrane composition is hard to emulate in model cell membranes [49]. Rodrigues et al. [12] demonstrated the concentration-dependent permeabilisation of Gm-treated murine melanoma cells (B16F10) by the extracellular release of the cytoplasmic enzyme lactate dehydrogenase (LDH, an estimate of cytosolic protein leakage) [12]. A concentration of at least 10 μM of Gm was needed to induce LDH release. A LDH-release assay was also used by Soletti et al. to show the membrane permeability of Gm in human neuroblastoma (SH-SY5Y) and rat pheochromocytoma cells (PC12) [17]. Compared to B16F10, LDH release was induced at concentrations <1 μM in these cell lines (see Figure 3C). It should be noted, however, that in the experiments with SH-SY5Y and PC12, LDH-release was assessed after incubation with Gm for 24 h, while in the experiments with B16F10 LDH-release was determined after 5, 15, and 30 min.

Based on the above-described leakage and permeabilisation experiments, both the pore-forming and carpet/detergent mechanisms have been suggested for Gm. While it has been proposed that the concentration of peptide required to induce membrane damage by the carpet mechanism is higher than for pore formation [50], at the concentrations used in the studies of Gm, both mechanisms are feasible. Domingues et al. suggested that Gm acts via a carpet mechanism [26,27,50] due to the sudden rupture of vesicles in leakage experiments, which was also observed previously for other AMPs [50]. The rupture was preceded by the accumulation of peptides on the membrane surface. On the other hand, Rodrigues et al. suggested that the clustering of fluorescently labelled Gm (Gm-Rh) on the surface of cancer cells is indicative of a pore-forming mechanism [12]. As both the carpet and pore mechanism can be preceded by the accumulation or clustering of peptide, this can neither exclude nor confirm a specific mechanism. Furthermore, it is possible that Gm-induced leakage in lipid vesicles proceeds via a different mechanism to that in cells [49,51]. Like with many other AMPs/ACPs, the exact lytic mechanism of action of Gm remains unclear. Without knowledge of the structure of peptide-membrane aggregates at the molecular level, and a more detailed understanding of the peptide-induced changes to membrane morphology, both the carpet and pore-forming mechanisms are plausible.

## 6. Membrane Binding and Peptide-Lipid Interactions

Leakage or permeabilisation necessitates membrane binding. Thus, a number of studies have investigated the binding of Gm and its variants to both model and cell membranes using a range of techniques [3,7,9,11,26,28].

**Figure 4:**
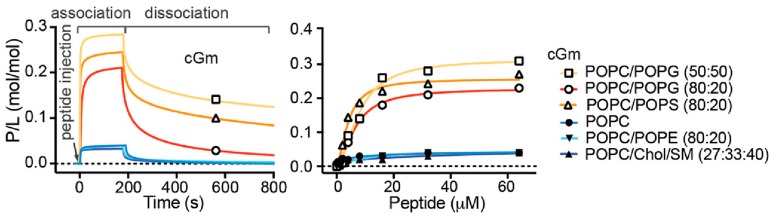
Binding of cyclic Gm (cGm) to model membranes of various lipid compositions determined by surface plasmon resonance (SPR) experiments. Left panel: sensorgrams obtained using a peptide concentration of 32 μM. Right panel: Dose-response binding curves for the different peptide concentrations. Response units were converted into moles of peptide and normalised for the amount of peptide (peptide-lipid ratio, P/L). Adapted with permission from Henriques et al. [9]. Copyright 2017 American Chemical Society.

Consistent with the increased leakage and permeabilisation of vesicles containing negatively charged lipids, Gm shows increased binding affinity for negatively charged membranes. SPR experiments have demonstrated that both Gm and cGm exhibit significantly increased binding to membranes composed of POPC/POPG (4:1) [3] and POPC/POPS (1:1) [9] compared to neutral membranes composed of POPC or POPC/POPE, as illustrated in Figure 4. This is related to the ability of Gm to specifically target bacterial membrane surfaces, which are negatively charged. Henriques et al. showed that cGm variants bind to the negatively charged liposaccharides (LPS) and lipoteichoic acid (LTA) found on the surface of Gram-negative and Gram-positive bacteria, respectively [9]. As described in Section 5, the addition of cholesterol to POPC vesicles resulted in a significant increase in the amount of Gm required to induce leakage [27]. SPR measurements did not reveal any significant difference in the binding of cGM to membranes composed of POPC/chol/SM (SM = sphingomyelin), compared to membranes composed of POPC or POPE/POPE (Figure 4). Cholesterol is known to increase lipid order while maintaining membrane fluidity, resulting in reduced permeability, which affects the ability of AMPs/ACPs to disrupt the membrane [20,30,52]. It is thus possible that the peptides exhibit the same degree of surface binding, as measured by SPR, while the addition of cholesterol affects the ability of the peptide to insert into, and eventually disintegrate, the membrane, which is measured in leakage experiments.

The binding of Gm and GmL to SDS micelles was confirmed by Moraes et al. using EPR spectroscopy. This study showed that, when bound to SDS micelles, the peptides exhibit significantly increased rotational correlation times, indicating a slower and more anisotropic motion [11]. To study the peptide-detergent interactions in molecular detail, Fazio et al. used NMR determinations with two types of paramagnetic agents to induce selective broadening of the resonances of amino acids that are either water exposed or buried at the water-detergent interface [7]. This approach was used to investigate the interaction of a GmL variant with SDS micelles. In this GmL variant, denoted [D-T^2,6,11,15,^ P^9^]-d-Gm, the C residues were replaced with T residues, and Q9 was replaced by P residues that promote the formation of β-strands and β-turns, respectively. This was combined with the substitution of all other residues except for the P9 and G16, by their D-isomers. Residues L5, T6, V12, T13, and Y14 in the β-strands showed much more peak broadening than residues at the termini, while peaks from residues at the β-turn were not affected by interactions with SDS. This indicates that Gm lies with its β-strands roughly parallel to the surface of the SDS micelle. Unfortunately, the experiments were not repeated with native Gm or GmL variants without the additional mutations, and it is thus not known whether that binding mode is unique to this variant or also observed in Gm variants with the β-turn-like fold. Also, in the absence of other structural data for lipid-Gm interactions, it is not possible to know whether the peptide would adopt a similar binding mode when bound to lipid vesicles or membranes.

Characterisation of the thermodynamics of interaction is critical to understanding the membrane-binding mechanism of Gm. Domingues et al. [25,26] and Mattei et al. [28] reported a series of ITC experiments examining the binding of Gm to POPC–POPG membranes with different fractions of POPG. In all studies, the ITC data was analysed using a surface partitioning model combined with the Gouy−Chapman theory. In contrast to the Langmuir model which assumes a fixed binding constant, this approach allows the binding constant (*K*_A_) to vary as a function of free peptide concentration, such that the apparent *K*_A_ can change as more and more peptide molecules bind to the surface. Secondly, the surface partitioning model also separates the binding affinity into two terms: an electrostatic term that describes the concentration of peptide close to the membrane, and a partition term that accounts for the adsorption/insertion of the peptide into the membrane. Table 3 summarises the thermodynamic parameters available for Gm binding to POPC–POPG membranes derived from ITC experiments. For an equimolar ratio of POPC and POPG, the *K*_A_ values obtained from ITC data analysed using a partition model are 5.0 μM^−1^ and 0.01 μM^−1^. This is equivalent to binding free energies (ΔG) of −22.1 kJ mol^−1^ and −12.6 kJ mol^−1^, respectively. There do not appear to be any apparent differences in the experimental protocols or conditions that would explain the considerable differences in *K*_A_ (and thus ΔG) in the two experiments, and the authors did not comment on them. The *K*_A_ obtained from analysis of ITC data using the Langmuir model was reported to be 5 μM^−1^, which is equivalent to −27.6 kJ mol^−1^. Consistent with the preference of Gm for binding to negatively charged membranes, the *K*_A_ for membranes with lower POPG content is reduced. However, due to the different fractions of POPG used, the binding constants from the two studies cannot be compared directly.

ITC data was also used to obtain the enthalpic (ΔH) and entropic (ΔS) contributions to ΔG, summarised in Table 3. Evaluation of these thermodynamic parameters shows that the binding of Gm to POPC–POPG membranes is exothermic. While Mattei et al. state that ”interaction of Gm with membranes is mainly enthalpy-driven, and the entropic contribution does not play a significant role”, the data in Table 3 shows that, depending on the membrane composition and study, entropy can make a substantial contribution to ΔG. In contrast to many helical AMPs, Gm adopts the same structure in water and when bound to a membrane surface, and consequently, the ΔH and ΔS associated with conformational changes is negligible. Thus, both the ΔH and ΔS contributions must arise predominantly from the gain of peptide-lipid interactions and loss of peptide-water interactions, as well as the release of water molecules from the water-lipid interface.

## 7. The Role of Charge and Hydrophobicity in the Mechanism of Gomesin

A commonly used strategy to understand the mechanism of action of biologically-active peptides is to prepare a series of peptide variants (also called mutants) with altered physico-chemical properties. Comparing the activity of peptide variants to the native peptide can then be used to establish a structure-activity relationship (SAR), which in turn could be used to design peptides with increased potency and selectivity towards bacterial or cancer cells. Nevertheless, as evident from the combined findings of a number of studies [6,9,10,11,18,28], establishing a SAR of Gm is not straightforward.

**Figure 5:**
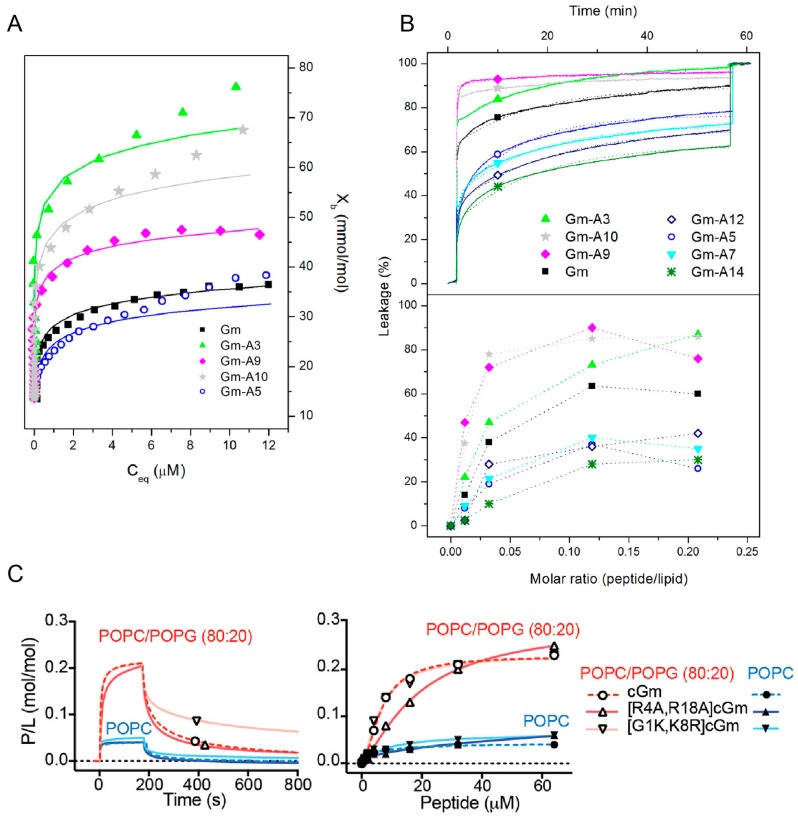
Membrane binding and leakage activity of Gomesin variants. (**A**) Binding isotherms showing molar fraction of bound peptide (Xb) as a function of free peptide concentration (C_eq_) obtained from ITC experiments of Gm and its variants binding to LUVs composed of POPC–POPG 7:3 (mol ratio); (**B**) Top panel: Kinetics of CF leakage from LUVs composed of POPC–POPG 7:3 (mol ratio) after addition of Gm and its variants at a peptide-molar ratio of 0.12 at room temperature (~23 °C). Bottom panel: percentage leakage as a function of peptide-lipid molar ratio; (**A**,**B**) were adapted with permission from Mattei et al. [28]. Copyright 2014 American Chemical Society; (**C**). Binding of cyclic Gm (cGm) and its variants to model membranes composed of POPC and POPC–POPG (4:1 molar ratio) from surface plasmon resonance (SPR) experiments. Left panel: sensorgrams from SPR experiments using a peptide concentration of 32 μM. Right panel: Dose-response binding curves from the different peptide-lipid concentrations. Response units were converted into moles of peptide and normalised for the amount of peptide. Adapted with permission from Henriques et al. [9]. Copyright 2017 American Chemical Society.

Most SAR studies of Gm have focused on investigating either the effects of changes in hydrophobicity or charge on its membrane binding, lytic, and cytotoxic activities. Mattei et al. designed a set of Gm variants with altered hydrophobicity/charge by replacing a single residue in the hydrophobic face (L5, Y7, V12, and Y14) or the polar/hydrophilic areas of the peptide (R3, Q9, R10) with an A residue (see also Figure 1) [28]. Replacing L5, Y7, V12, or Y14 with A reduces the hydrophobicity of the peptide, while replacing the charged or polar R3, Q9, R10 with A increases hydrophobicity (Table 4). The membrane binding of these variants to LUVs composed of POPC–POPG (7:3 mol ratio) was measured using ITC, while their ability to permeabilise said LUVs was assessed using CF leakage experiments. Comparison of native Gm and its variants revealed that the more hydrophobic peptides generally have higher membrane binding affinities (Figure 5A and Table 4) and higher percentage of leakage (Figure 5B). This indicates that hydrophobicity plays an important role in the membrane-binding and lytic activity of Gm. The extent of this effect, however, depends on the position of the residue that is mutated. For example, while replacing the hydrophobic residues Y7, V12, or Y14 with an A residue reduced membrane binding to below the detection limit of ITC, the variant with the same mutation on L5 still showed some degree of membrane binding (albeit reduced, compared to native Gm). Similarly, the increase in membrane binding affinity is much larger when replacing R3 with A compared to R10. A decrease in binding affinity and leakage for peptides that are less hydrophobic can be rationalised by the fact that for the peptide to disrupt the membrane, it must interact with its hydrophobic core. Peptides with a reduced hydrophobic character might lose that ability. In contrast, the increased affinity of the R3 and R10 variants is somewhat counterintuitive. While these variants follow the hydrophobicity trend, replacing R3 or R10 with A reduces the +6 charge of native Gm to +5, yet these variants show increased binding to negatively charged membranes in comparison to native Gm (see Figure 5A and Table 4).

In this respect, Henriques et al. studied the membrane binding, leakage, and cytotoxic activity of a range of cGm variants, including some that alter the charge of the peptide [9]. Compared to the +6 charge of cGm, the variants cGm[G1K,K8R] and cGm[R4A,R18A] have a charge of +8 and +4, respectively. Based on the ‘hydrophobicity trend’ alone, cGm[G1K,K8R] with its reduced hydrophobicity should show decreased binding, while the increase of hydrophobicity in cGm[R4A,R18A] should increase binding. However, SPR experiments with membranes composed of POPC–POPG (4:1 molar ratio) reveal that both peptides have similar binding affinities (Figure 5C). The only difference in their binding is that cGm[R4A,R18A] has a slower association rate, consistent with its reduced charge, while cGm[G1K,K8R] has a reduced dissociation rate (Figure 5C). The leakage activity of both of these variants is also inconsistent with the ‘hydrophobicity trend’. cGm[R4A,R18A] showed a 4-fold reduction in its ability to induce leakage in LUVs composed of POPC–POPG (4:1 molar ratio), while cGm[G1K,K8R] shows a 4-fold increase in leakage. Another example is the truncated variant cGm(2−15), where pE1 and R16-G17-R18 were removed. This peptide has a reduced charge, and thus, increased hydrophobicity, but the variant shows reduced anti-microbial activity, implying a reduced leakage activity [10]. Finally, for cancer cells, an increase in membrane binding and lytic activity does not necessarily translate into increased cytotoxic activity. The Gm-R3A mutant has a higher binding affinity and induces more leakage than Gm [28], but it shows no cytotoxic activity against DFTD [18] or melanoma cells (Ikonomopoulou et al., unpublished).

These studies with Gm variants indicate that while hydrophobicity is important for the membrane binding and lytic activity of the peptide, there is no general trend that is predictive. The specific activity of a variant is more likely to result from the fine balance between the overall hydrophobicity and charge of the peptide, as well as the spatial distribution of hydrophobic/non-polar and polar/charged residues on the surface of the peptide, as this will dictate the extent to which the peptide can bind to and insert into the membrane. In some cases, membrane-binding and lytic activity on model membranes might not translate into cytotoxic activity [51]; this highlights the complexity of membrane-disrupting mechanisms of AMP/ACPs.

## 8. Intra-Cellular Effects

A number of studies have shown that, in addition to causing cell death by permeabilisation, many AMPs and ACPs also exert cytotoxicity via several intracellular mechanisms (see Gaspar et al. [19] and references therein). In the case of Gm, it has been shown that it affects programmed cell death [13,17,18,33,54], expression levels of cell cycle proteins [18], generation of reactive oxygen species (ROS) [17,18], and the intracellular levels of Ca^2+^ [16,17,33].

Experiments using CHO cells [33], B16F10 mouse melanoma cells [12,13], and K562 cells [14] have shown that Gm can be internalised at concentrations below the ones required for permeabilisation. Once inside the cell, the peptide can interfere with necrotic or apoptotic pathways in a cell-type specific manner. Experiments with K562 cells showed that cell death was primarily induced via apoptosis [16]. On the other hand, necrosis seems to be the dominant pathway in SH-SY5Y [17] and B16F10 [12] cells, neither of which showed any signs of apoptosis. Similarly, Gm induces necrosis in DFTD4 cells [18]. It is thus likely that the detailed mechanism is cell-type specific.

In some cell types, Gm-induced cell death is associated with changes of intracellular Ca^2+^ levels. For example, Paredes et al. [33] showed that in CHO cells membrane permeabilisation was preceded by an elevation of cytosolic Ca^2+^ concentrations. This was accompanied by a release of Ca^2+^ from the endoplasmic reticulum followed by a disturbance of the cell mitochondria and lysosomes. In another study, the same authors also showed increases of Ca^2+^ levels in K562 cells after treatment with Gm [16]. The role of Ca^2+^ in Gm-induced cell death was also demonstrated in SH-SY5Y and PC12 cells [17]. Ca^2+^ influx increase evoked by Gm was significantly reduced when pre-treating cells with nimodipine, a blocker of l-type voltage-dependent calcium channels. Furthermore, the increase of Ca^2+^ levels activated a number of signalling pathways resulting in the increase of ROS. The authors concluded that the Gm-induced cytotoxicity of human neuroblastoma cells involves “calcium entry through L-type voltage-dependent calcium channels”, activation of kinase signalling pathways, and generation of ROS. Increased generation of ROS was also shown for DFTF4 cells treated with Gm and its variants [18]. The same study also showed the presence of other hallmarks of cellular stress in Gm-treated cells, including changes in the expression of cell cycle inhibitory proteins p53, p27, and p21, as well as diminished mitochondrial membrane potential that correlated with a significant reduction of the G0/G1 cell cycle phase. All of these experiments clearly show that, in addition to membrane permeabilisation, Gm can promote cell death through a range of cell-type specific mechanisms.

## 9. Conclusions

Since its original discovery and isolation, a number of studies have confirmed the cytotoxic activity of Gm against a range of clinically-relevant microbes as well as cancer cells. The therapeutic potential of Gm to treat candidiasis and as an anti-cancer agent to treat melanoma has been confirmed in mouse models. The biophysical characterisation of Gm and Gm variants has shown that the β-sheet structure is required for its anti-microbial and anti-cancer activity, as well as its serum stability. Attempts to reduce the haemolytic activity of Gm demonstrated that this is usually accompanied by reduced anti-microbial and anti-cancer activity. It is, however, possible to improve the activity against target cells while not altering haemolytic activity, thus improving the overall therapeutic index of the peptide. Studies of the membrane binding and lytic activities of Gm and Gm variants have demonstrated that both hydrophobicity and charge are important for the cytotoxic activity of Gm. Improving cytotoxic activity or selectivity is, however, not as simple as increasing hydrophobicity and/or charge, but rather, more a case of finding an optimal distribution of hydrophobic/non-polar and polar/charged residues on the surface of the peptide. Without a more detailed understanding of the peptide-lipid interactions at the molecule level, a rational design of variants with improved cytotoxic activity or selectivity remains a non-trivial task.

In the search for establishing a SAR and finding variants of improved cytotoxic activity, it has also become clear that the detailed mechanism of Gm depends on a number of factors, including peptide concentration and cell type. At low concentrations, Gm can alter programmed cell death, expression levels of cell cycle proteins, and alter intra-cellular levels of Ca^2+^. The details of the pathways and processes involved are cell type-specific. At higher concentrations, Gm causes cell death via membrane permeabilisation. Even if the presence of negatively charged lipids such PS on cancer cells or LPS on Gram-negative bacteria can explain the selectivity of Gm over healthy mammalian cells, it is not clear why Gm shows marked differences in activities across different cancer cell lines or bacterial strains. Further research is needed to establish whether these are based on differences in membrane composition, intra-cellular effects, or both. Understanding the effect of specific lipids on the membrane-disrupting activity and cell-type-specific selectivity of Gm (and other AMPs/ACPs) will require the systematic study of vesicles or membranes of various lipid compositions, as well as the use of more realistic model membranes that better mimic healthy mammalian cells and cancer or bacterial cells. Finally, to establish the clinical use of Gm to treat microbial infections or cancer, more pre-clinical studies using animal models are required [19,55].

## Figures and Tables

**Table 1:** Anti-microbial activity of Gm for the most commonly tested microorganisms. MIC values from different studies are combined unless they are significantly different from each other.

Microorganism	MIC (µM)	Reference
*Escherichia coli*		
*E. coli SBS 363*	0.32–1.6	[8,11]
*E. coli 1106*	0.8–1.6	[1]
*E. coli ATCC 25922b*	4	[9]
*E. coli DH5a*	3.4	[3]
*Pseudomonas aeruginosa*		
*P. aeruginosa ATCC 27853b*	8	[9]
*P. aeruginosa* *	1.6–3.15	[1]
*Staphylococcus aureus*		
*S. aureus ATCC 25923b*	32	[9]
*S. aureus* *	1.6–3.15	[1]
*Klebsiella pneumoniae*		
*K. pneumoniae ATCC 700603b*	32	[9]
*K. pneumoniae* *	3.15–6.25	[1]
*Candida albicans*		
*C. albicans MDM8*	0.15–1.28	[8,11]
*C. albicans ATCC 90028(d)*	8–16	[9,34]
*C. albicans 78*	5.5	[34]
*C. albicans* *	0.15–0.30	[1]
*Cryptococcus neoformans*		
*C. neoformans ATCC 208821*	0.5–1.0	[9]
*C. neoformans* *		[1]

* Strain number not reported in the referenced study.

**Table 2:** Anti-cancer activity of Gm against the most commonly tested cell lines, reported as IC_50_, EC_50_ and/or CC_50_ ± standard deviations. Numbers is brackets indicate a 95% confidence interval.

Cell Line	IC_50_ (μM)	EC_50_ (μM)	CC_50_ (μM)	Reference
**Skin cancer**				
B16 F10 (murine)	7			[13]
	3.58 (2.76–4.41)			[12]
MM96L (human)	5.5 ± 1.1		3.7 ± 0.2	[3,9]
SKMel 19 (human)	2.35 (1.69–3.00)			[12]
A2058 (human)	1.36 (0.29–3.02)			[12]
**Breast cancer**				
SKBr3 (human)	2.87 (1.30–4.45)			[12]
	19.8 ± 1.2			[15]
**Cervical cancer**				
HeLa (human)	72.7 ± 2.5			[3]
	25.6 ± 1.3			[15]
	8.13 (6.00–10.25)			[12]
			54.1 ± 5.0	[9]
**Leukaemia (human)**				
K562		6.2 ± 1.3	3.8 ± 0.3	[9,14]
Kasumi		10.6 ± 0.2		[14]
ARH77		6.7 ± 0.8		[14]
CCRF-CEM		13.1 ± 1.7		[14]
**Devil facial tumour disease**				
DFTD1,2,4		20.4, 12.29, 20.41		[18]

**Table 3:** Thermodynamic parameters determined by isothermal titration calorimetry (ITC) measurements of the binding of Gomesin to membranes composed of POPC with different fractions of POPG. Data from binding isotherms was fitted using a Langmuir model or a partition model.

Membrane Composition (Mol Ratio)	Binding Model	K_A_ (μM^−1^)	K_D_ (1/K_A_) (μM)	ΔG (kJ mol^−1^)	ΔH (kJ mol^−1^)	TΔS (kJ mol^−1^)
POPC–POPG (1:1) [25]	Langmuir	5	0.2	−27.6	−33.4	−5.8
POPC–POPG (1:1) [25]	Partition	0.01	100	−12.6	−32.6	−20.0
POPC–POPG (1:1) [26]	Partition	0.5	2	−22.1	−29.2	−7.1
POPC–POPG (3:1) [26]	Partition	0.01	100	−12.6	−23.4	−10.8
POPC–POPG (7:3) [28]	Partition	125 × 10^−3^	8.0 × 10^3^	−2.0	−33.0	−31.0

**Table 4:** List of Gm variants with increased or decreased hydrophobicity, their charge and thermodynamic parameters (*K*_A_, ΔG, ΔH and ΔTS) for binding to POPC–POPG membranes obtained from ITC. ΔΔG _w__⟶__b_ is the difference in hydrophobicity between the variant and WT Gm based on an experimentally determined residue-based hydrophobicity scale [53]. Negative values indicate a peptide that is more hydrophobic. Table adapted from Mattei et al. [28]. The binding constant *K*_A_, and thus ΔG and TΔS could only be obtained for variants with a ΔH < −20 kJ mol^−1^.

Peptide	Charge	ΔΔG_w⟶b_ (kJ mol^−1^)	ΔH (kJ mol^−1^)	ΔG (kJ mol^−1^)	*K*_A_ (mM^−1^)	TΔS (kJ mol^−1^)
R3A	5	−2.7	−24.2	−36.8	50	11.7
R10A	5	−2.7	−40.1	−32.2	8	−8.6
Q9A	6	−1.7	−37.6	−30.9	5	−7.2
**Gm (WT)**	6	0.0	−33.0	−21.7	0.125	−11.6
V12A	6	0.4	−6.7	-	-	-
L5A	6	3.3	−26.8	−19.6	0.05	−7.5
Y7A	6	4.6	−6.7	-	-	-
Y14A	6	4.6	−4.2	-	-	-
